# Bak and Mcl-1 are essential for Temozolomide induced cell death in human glioma

**DOI:** 10.18632/oncotarget.1642

**Published:** 2014-01-01

**Authors:** Catherine Gratas, Quentin Séry, Marion Rabé, Lisa Oliver, François M. Vallette

**Affiliations:** ^1^ Centre de Recherche en Cancérologie Nantes Angers, UMR INSERM 892 / CNRS 6299; ^2^ Université de Nantes, Nantes F-44000, France; ^3^ CHU de Nantes, Nantes F-44805, France; ^4^ Institut de Cancérologie de l’Ouest-Centre René Gauducheau, St Herblain F-44805, France

**Keywords:** temozolomide, glioma, apoptosis, Bcl-2 family

## Abstract

Temozolomide (TMZ) is an alkylating agent used for the treatment of glioblastoma multiforme (GBM), the main form of human brain tumours in adults. It has been reported that TMZ induced DNA lesions that subsequently trigger cell death but the actual mechanisms involved in the process are still unclear. We investigated the implication of major proteins of the Bcl-2 family in TMZ-induced cell death in GBM cell lines at concentrations closed to that reached in the brain during the treatments. We did not observe modulation of autophagy at these concentrations but we found an induction of apoptosis. Using RNA interference, we showed that TMZ induced apoptosis is dependent on the pro-apoptotic protein Bak but independent of the pro-apoptotic protein Bax. Apoptosis was not enhanced by ABT-737, an inhibitor of Bcl-2/Bcl-Xl/Bcl-W but not Mcl-1. The knock-down of Mcl-1 expression increased TMZ induced apoptosis. Our results identify a Mcl-1/Bak axis for TMZ induced apoptosis in GBM and thus unravel a target to overcome therapeutic resistance toward TMZ.

## INTRODUCTION

*Glioblastoma multiforme* (GBM), the most common form of brain cancer in the adults, are highly resistant to current treatments. The 2-year survival rate remains low as most of the tumours recur despite the aggressive treatment combining surgery, radio and chemotherapy [1]. Recently, the introduction of temozolomide (TMZ) in association with radiotherapy in the treatment of GBM has produced a small but significant over all survival in a subset of patients [2]. The cytotoxicity of TMZ is mainly mediated by the generation of O^6^methylguanine in DNA, which triggers cell cycle dependent DNA damage and ensuing cell death [3]. The expression of O^6^-methylguanine-DNA methyl transferase (MGMT) limits TMZ therapeutic efficacy by removing O^6^-methylguanine [4].

So far, the only prognostic/predictive factor for longer survival after TMZ treatment is the partial or total silencing of MGMT through methylation of its gene promoter which favours TMZ induced major defects in DNA repair [4]. However, both in vitro and in vivo, cell death triggered by TMZ at therapeutic doses is still low even in MGMT silenced tumours [3]. This means that other factors, which are currently not known, are probably involved in GBM resistance. The precise mechanism of cell death triggered by TMZ, is still subject to controversial discussion and studies of the association of TMZ with other drugs indeed revealed a complex array of mechanisms implicated in TMZ cytotoxicity [5]. It has been reported that TMZ through the production of O^6^-methylguanine was inducing apoptosis [6]. However, the actual apoptotic mechanism triggering and amplifying the response to TMZ is still unknown as contradictory results have been published (see for example references 7 and 8). Moreover the importance of apoptosis has been challenged by others who observed predominantly an induction of autophagy after TMZ treatment of glioma cell lines [9]. Apoptosis and autophagy are both cell death programs but the latter is also an essential component of cellular survival mechanism which plays an important role in cancer [10]. TMZ was although shown to induce mitotic catastrophe in p53-deficient glioma cells [11]. Additional signalling pathways have been associated with TMZ cytotoxicity such as G2/M check point integrity [12], up-regulation of miR-21 [13], induction of senescence [11, 14], and elevated AMP-activated Protein Kinase [15].

However, in most of these studies, the concentrations of TMZ used were far greater than that achieved during GBM treatment (i.e.≥ 50 μM) and thus did not necessarily reflect TMZ actual toxicity in patients. We thus decided to evaluate the implication of proteins of the Bcl-2 family at TMZ therapeutic doses [16] as members of this family are central in the control of both apoptosis and autophagy downstream of DNA damage [17-21].

## RESULTS

### Effect of TMZ on the induction of cell death and cell cycle in glioma cell lines

**Figure 1:**
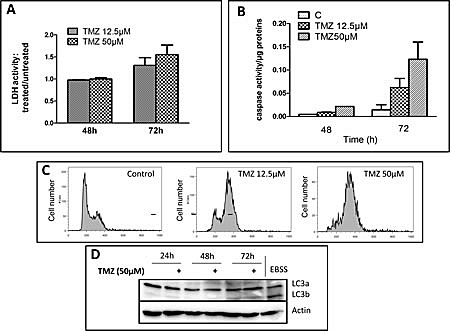
TMZ induced apoptosis and G2 cell arrest at therapeutic concentrations in U251 cells U251 cells were treated with 12.5 μM or 50 μM TMZ. (A) LDH activity was measured in the supernatant at indicated times. Data are expressed as mean +/- SD. (B) DEVDase activity was measured after 48 and 72h TMZ exposure. (C) Cells were stained with iodide propidium and cell cycle analysis was performed at 48h post-TMZ exposure. (D) Western Blot showing no LC3 cleavage in response to TMZ in U251 cells. EBSS medium was used as positive control of autophagy and both LC3-I and LC3-II are present. Each experiment was performed at least 3 times independently.

We used a human glioma cell line which does not express MGMT, U251, but with a mutant p53 status. We used TMZ concentrations up to 50 μM, concentration closed to the AUC in brain (31+/-6.2μM/l/h) as described by Ostermann et al. [16]. To analyze cell death after TMZ treatment, we used a LDH assay (see materials and methods). The LDH activity in the supernatant was only moderately increased after incubation for 3 days with both TMZ concentrations (i.e. 12.5 and 50 μM) (Figure 1A). In cells treated for up to 72 hrs in the continuous presence of TMZ, apoptosis was quantified every 24 hrs by measuring caspase 3 activity (i.e. DEVDase activity) as described in materials and methods. As showed in Figure 1B, caspase activity was only increased after 72 hrs at 12.5 and 50 μM TMZ. In presence of the inhibitor of DEVDase activity (DEVD-CHO), no caspase activity was observed (Figure S1). Block in G2/M has been proposed as the main effect of TMZ on glioma [11]. As shown in Figure 1C, TMZ induced a block in G2/M in U251 cell line at both concentrations with similar amplitude. At higher dose (i.e. 100 μM), it has been reported that TMZ induced autophagy, but not apoptosis in malignant glioma cells [9]. Autophagy can be monitored by the analysis of the conversion of soluble LC3-I to lipid bound LC3-II which is associated with the formation of autophagosomes. We used LC3 antibodies to detect autophagy. As shown in Figure 1D, the incubation of U251 with 50 μM TMZ did not trigger autophagy. Similar results were obtained with other markers of autophagy such as autophagosome labelling with monodansylpentane (MDH) and degradation of p62/ SQSTM1, a LC3-II partner (data not shown). From these results, we conclude that apoptosis would prevail over autophagy at TMZ concentrations inferior to 100 μM.

### Role of Bcl-2, Bcl-Xl and Mcl-1 in U251 response to TMZ

**Figure 2:**
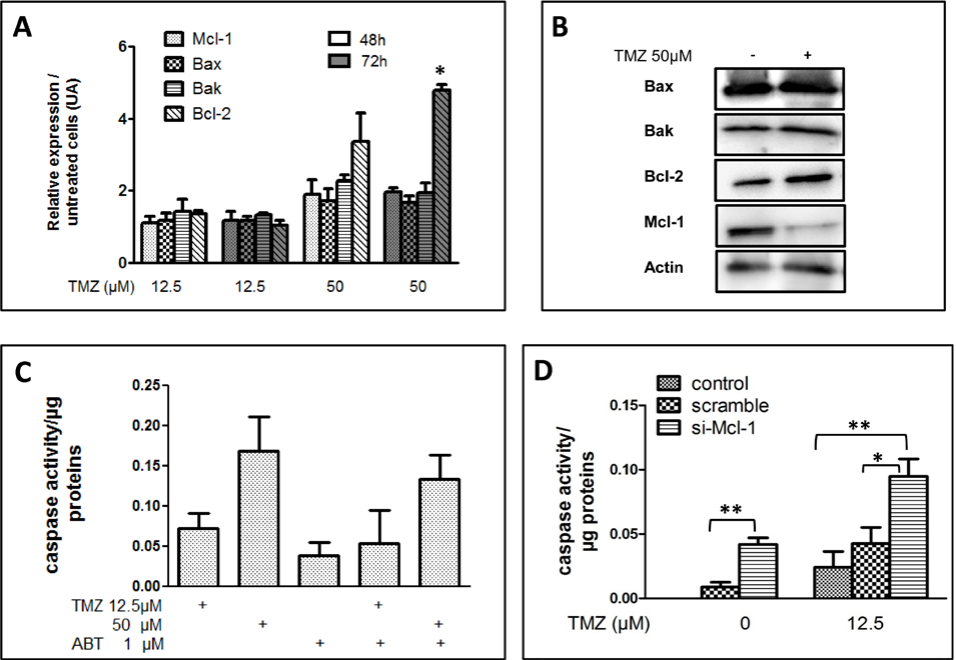
Influence of TMZ on the expression of key members of the Bcl-2 family (A) RNA expression of Mcl-1, Bcl-2, Bax and Bak in U251 cells treated with 12.5 μM and 50 μM TMZ for 48h or 72h was evaluated by qPCR. Data are expressed as mean +/- SD. (B) Immunoblots analyses of Bax, Bak, Bcl-2 and Mcl-1 in U251 treated or not with 50 μM TMZ for 72h. The graph is illustrative of one of three independent experiments. (C) DEVDase activity measured in proteins extracts, 72h post-treatment of cells by TMZ (12.5 or 50 μM) plus or minus ABT 1 μM. (D) DEVDase activity measured in protein extracts of untreated cells or cells transfected with si-RNA, scramble or directed against Mcl-1, and treated for 48h with 12.5 μM TMZ. Data are expressed as mean +/- SD, n=3.

Bcl-2 family members are regulators of apoptosis [18]. We thus analyzed the expression of several key members of the Bcl-2 family after TMZ treatment at 12.5 or 50 μM: namely Mcl-1 and Bcl-2, two anti-apoptotic members of the Bcl-2 family and Bax and Bak, two pro-apoptotic members of the Bcl-2 family. As illustrated in Figure 2A, the expression of mRNA encoding for Bcl-2, quantified by Q-PCR (see materials and methods) was significantly increased in cells surviving TMZ treatment (i.e. living cells) at 50 μM after 48 and 72 hrs. On the other hand, little or no differences were observed for the expression of Mcl-1, Bax and Bak after the treatment (Figure 2A). Next, we examined the expression of these proteins by immunoblots. As illustrated in Figure 2B, TMZ did not induce any change in the expression of Bax and Bak at the protein level. On the other hand, the expression of Bcl-2 was increased and that of Mcl-1 considerably reduced during the treatment. Several small molecules have been selected on the basis of their anti-Bcl-2 activity and among them ABT-737 has been shown to be a more potent inhibitor of Bcl-2/Bcl-Xl/ Bcl-W but not of Mcl-1 [22]. The treatment of U251 cell line with ABT-737 in the presence of TMZ, did not affect apoptosis significantly (Figure 2C), ruling out the involvement of Bcl-2/Bcl-Xl/ Bcl-W in the inhibition of TMZ induced apoptosis. To examine the importance of Mcl-1 in TMZ induced cell death, we treated the cells with Si-RNAs designed to inhibit the expression of Mcl-1 (Figure S2A). As illustrated in Figure 2D, the silencing of Mcl-1 enhanced apoptosis induced by TMZ in U251 cell line even at the lowest concentration (i.e. 12.5 μM), when the amount of Mcl-1 was not affected by the drug (Figure S2A). Of note, at the higher concentration (i.e. 50 μM), all cells with silenced Mcl-1 were killed by the TMZ treatment (data not shown).

These results showed for the first time that Mcl-1 play an important role in TMZ induced apoptosis.

### Role of Bax and Bak in U251 response to TMZ

**Figure 3:**
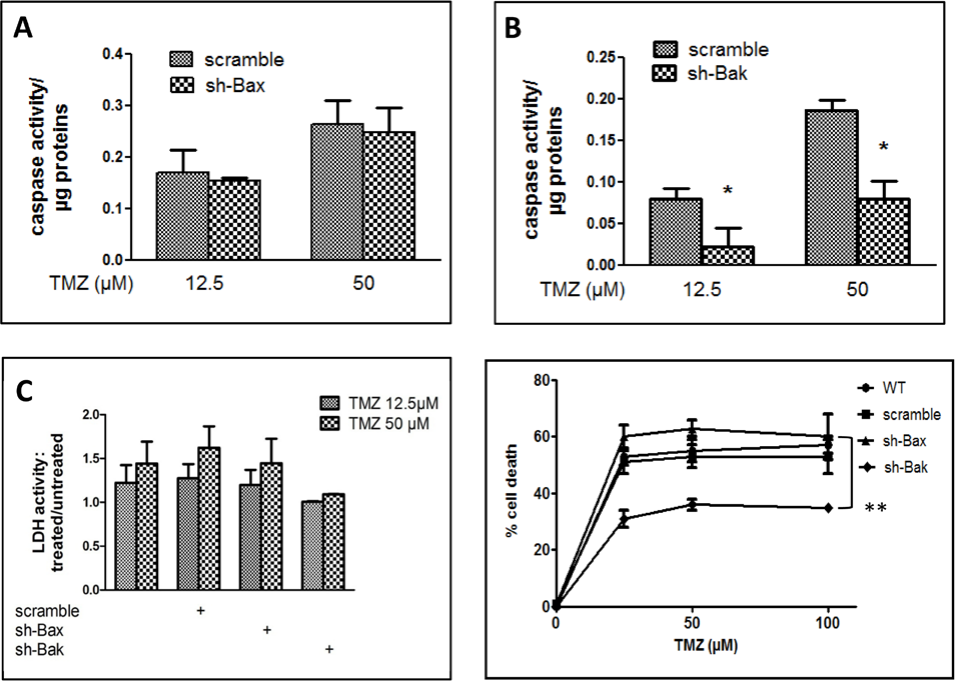
TMZ induced apoptosis in U251 cells via the pro-apoptotic protein Bak (A) DEVDase activity measured in U251 cells infected with sh-scramble or sh-Bax and treated with 12.5 or 50 μM TMZ for 72h. (B) DEVDase activity measured in U251 cells infected with sh-scramble or sh-Bak and treated with 12.5 or 50 μM TMZ for 72h. (C) Measure of the LDH activity in the supernatant of U251 infected with scrambled, sh-Bak or sh-Bax treated with TMZ. (D) Data are expressed as mean +/- SD, n=3. (D) MTT assay with U251 cells infected with scramble, sh-Bak or sh-Bax, and treated with 25, 50 or 100 μM TMZ for 72h. Data are expressed as mean +/- SD, n=3.

The expression of Bax and Bak did not appear to be modulated by TMZ treatment. However these proteins, depending on the nature of the death inducing agent, do not exhibit similar sensitivities or implications during apoptosis [23]. To examine the role each of these proteins play in TMZ response we knocked down the expression of Bax or Bak in U251 by RNA interference (Figure S2B and C). When Sh-Bax treated cells were incubated with TMZ, we observed little or no influence on induced apoptosis (Figure 3A). Conversely, the knock down of Bak expression significantly decreased the induction of caspase activity by TMZ treatment (Figure 3B). Of note, in sh-Bak treated cells, a slight decreased in necrosis related cytotoxicity was observed as shown by LDH activity measurements (Figure 3C). The effect of Bak on apoptosis was not dose dependent (Figure 3D) and no effect on cell cycling was observed in TMZ treated control and Sh-Bak or Bax U251 cells (data not shown).

**Figure 4:**
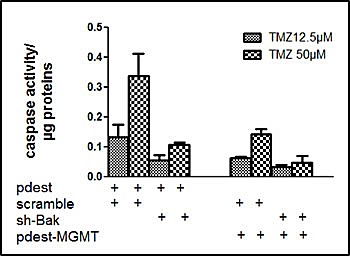
TMZ induced apoptosis in U251-MGMT positive cells via Bak protein U251 cells were transfected with a control plasmid pDEST 12.2 or a vector pDEST-MGMT in presence or in absence of sh-Bak. DEVDase activity was measured in protein extracts after 72 h exposure to 12.5 or 50μM TMZ. Results are expressed as mean +/- SD, n=3

It has been shown that in TMZ treated glioma cell line, that repair of O^6^-methylguanine by MGMT prevented apoptosis (6). We thus expressed MGMT in U251 cell line (Figure S3) which otherwise does not express this enzyme. The expression of MGMT decreased the response of U251 cells to TMZ as expected (Figure 4). However, the expression of MGMT in U251 did not change the specific role of Bak in TMZ toxicity (Figure 4).

### LN18 exhibited similar Bak/Mcl-1 specificity toward TMZ induced apoptosis

**Figure 5:**
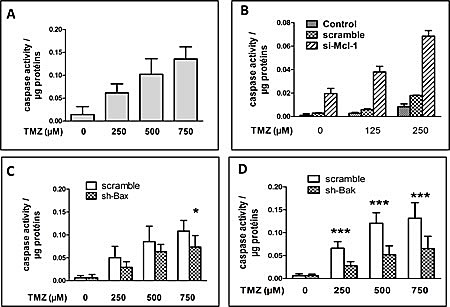
TMZ induced apoptosis in LN18 is also Mcl-1 and Bak dependent (A) DEVDase activity measured after 72h TMZ exposure at 250, 500 or 750 μM. Each experiment was performed at least 3 times independently. (B) DEVDase activity measured in proteins extracts of untreated cells or cells transfected with scramble or si-Mcl-1, and treated for 72h with 125 or 250 μM TMZ. Data are expressed as mean +/- SD, n=3. (C and D) DEVDase activity measured in LN18 cells treated with scramble, sh-Bak or sh-Bax and treated or not with 250, 500 and 750 μM TMZ for 72h

We analyzed the implication of Bak in TMZ induced apoptosis in LN18 cells; a PTEN/MGMT positive cell line. As shown in Figure 5A, the induction of apoptosis required a concentration of TMZ superior to that observed for U251 cells (i.e. 250 to 750 μM). The knock down of the expression of Mcl-1 (Figure S4A) has a deep influence on TMZ induced apoptosis (Figure 5B) while that of Bax (Figure S4B) had very little influence on cell survival except for at the highest concentration (i.e. 750 μM) (Figure 5C). On the other hand, the decrease in the expression of Bak (Figure S4C) inhibited significantly, TMZ (at all concentrations) induced apoptosis (Figure 5D).

## DISCUSSION

The introduction of TMZ/ irradiation regimen in the treatment of gliomas has significantly improved the outcome of patients [1-2]. However, benefit in terms of survival is mainly restricted to some patients exhibiting MGMT promoter methylation [4, 24, 25]. Indeed, the MGMT promoter could be predictive or prognostic for a sub-group of gliomas that are exhibiting specific gene methylation profile and/or more sensitive to cell death induction as suggested by our previous studies [26]. We have particularly shown that a DNA-methylating factor, such as the folinic acid, induced limited cellular proliferation and increased the sensitivity to TMZ-induced apoptosis in glioma cells [27]. Of note, among silenced genes, several were implicated in apoptosis such as survivin, and Bcl-W and others such as MGMT specifically implicated in DNA repair and response to TMZ [27].

Despite numerous works, the mechanisms by which TMZ induce cell death in cancer cells remain elusive. It has been proposed that the main features of TMZ cytotoxicity was associated with induction of cell death or cell cycle arrest [4]. In the present study, we show that TMZ at a concentration equal or inferior to that achieved during patients treatment [16], induced a type of cell death which is quantitatively and qualitatively different from that described by others at higher concentrations (i.e. >100 μM). As illustrated in Figure 1, apoptosis was always limited in treated glioma cell lines. On the other hand, no or little effect on autophagy was observed at these TMZ concentrations (Figure 1). However, we observed that TMZ induced cell death was a mixture of apoptosis and another cell death programme which remain to be identified.

Since the Bcl-2 family members are central to most cell death programmes, we have investigated the roles of some of its key members in TMZ induced cytotoxicity. It has been reported that TMZ at high concentrations (i.e. > 100 μM) induced a change in the Bax: Bcl-2 ratio which was instrumental to its cytotoxicity through enhancement of apoptosis and/or autophagy [6-9]. In this study, we did not observe any change in the expression of Mcl-1, Bax or Bak upon TMZ treatment at lower concentrations. Pharmacological inhibition of Bcl-2/Bcl-Xl/ Bcl-W by ABT-737 did not affect cell death under our conditions while the knock down of the expression of Mcl-1 affected apoptosis (Figure 2). Similarly, the knock down of Bax expression did not affect TMZ induced apoptosis while that of Bak inhibited this cell death programme. This feature was conserved in the presence of MGMT (Figure 4). Quite remarkably, such a difference was not found for necrosis-like cell death quantified by the release of LDH where Bax and Bak appeared to play similar roles (Figure 1). However, neither Bax nor Bak have an influence on autophagy upon TMZ treatment (data not shown). We, and others, have shown distinct roles for Bax and Bak especially in linking chemotherapeutic drugs or death receptor pathways to the mitochondrial apoptosis signalling cascade [23, 28-30]. These differences have been associated with the extent of the inhibitory effect of Mcl-1 on Bak [29]. Interestingly, recent results suggest that the alkylating DNA-damage agent N-methyl-N’-nitro-N-nitrosoguanidine (MNNG) induced cell death through a caspase independent but Bax dependent pathway called necroptosis [31]. Further investigations are necessary to verify if TMZ under our conditions induced both a Bax dependent necroptosis and a Bak dependent apoptosis. Similarly, the implication of members of the Bcl-2 family should be examined in the new therapeutic targets that have been recently described [32-36].

Altogether, we have identified in this work a Mcl-1/Bak axis implicated in TMZ induced apoptosis in two different glioma cell lines. These cell lines differ in their PTEN and MGMT status, two features that influence their response to TMZ. We also show that upon TMZ treatment Mcl-1 expression is not modified at the transcriptomic level but at the protein level.This suggests that targeting Mcl-1 either by competitive inhibitors of its interaction with Bak or by promoting its degradation is a potentially important target in glioma.

In addition, our results suggest that Bax is not involved in the apoptotic pathway (i.e. activation of caspase 3) but is nonetheless associated with cytotoxicity at concentrations that are likely to be reached during treatment of GBM patients. Thus a better understanding of this non canonical cell death programme induced by TMZ might be important to improve the efficacy of GBM patient's treatment.

## Materials and Methods

### Reagents and Materials

All reagents were from Sigma except for the Temozolomide (TMZ) obtained from Interchim (Paris, France). The stock solution of TMZ in DMSO at 100mM. Aliquots were frozen at −20°C and dilutions to the appropriate concentrations were done in culture medium just before the experiment. The following antibodies were used: Mcl-1 (sc-819 Santa Cruz), Bax (2D2, Sigma), Bak (556396- BD-Pharmingen USA), SQSTM1/p62(5114-Cell Signaling) and LC3B (2775- Cell Signaling), β Tubulin (T0198-Sigma), αActin (clone C4, MAB1501, Millipore). Secondary mouse and rabbit antibodies were from Jackson ImmunoResearch (UK). For MGMT transfection the plasmid pDEST 12.2 was obtained from In Vitrogen, Life technologies (CA) and the MGMT cDNA from ATCC, clone MGC-5186.

### Cell culture

U251 cell line and its derivatives were grown in high-glucose Dulbecco Modifed Medium enriched with 10% fetal calf serum, antibiotics and glutamine (Life technologies, Carlsbad, CA). Cells were maintained in 5%CO2 incubator at 37°C.

### Cell cycle analysis

Cells were plated in 6 well-plates, treated with TMZ for the respective time and collected by trypsinization. After fixation in 70% alcohol, cells were stained with propidium iodide (8μg/ml) in presence of RNAseA-DNAse free (Amresco, Solon, OH). Cells were then analysed on a BD-Facscan and the Cell Quest software (BD Biosciences, Franklin Lakes, NJ).

### Cell viability assays

Cell viability was assayed by MTT [37]. Cells were seeded in 96 well plates. Next day drugs were added in 100μL at the indicated concentration and plates were incubated for 72h. Controls contained drug vehicles. MTT 0,5mg/ml was then added and after 4 h at 37°C, medium was removed and formazan precipitates were dissolved in DMSO. Optical density was read at 570 nm on a microplate reader (Molecular Device, CA).

### Knockdown experiments

### Lentivirus

Cells were transduced with MISSION^®^ shRNA Lentiviral Particles against Bax (SHVRS NM_004324.3) and Bak (SHVRS NM_001188) as recommended by the manufacturer (Sigma-Aldrich,Saint-Louis, MO). Five sequences were used and after cell selection with puromycin (2μg/ml) the one with the best gene inhibition was selected for experiments. Scramble sequences (SHC001V; SHC002V) were used as negative controls.

### Si RNA

To inhibit Mcl-1, Silencer si RNAs (120644-Ambion, Applied Systems) were transfected in U251 cell line, with Lipofectamine RNAi Max (Life technologies) according to the recommended protocol. Final concentration of si RNA was 10μM. si-RNA-A, sc-37007, a non targeted si-RNA (Santa-Cruz, CA), was used as negative control. After 48h cells were collected for protein extraction.

### Protein extraction

Cell lysis was done in ice-cold RIPA buffer containing protease inhibitors, for 20 min. After centrifugation at 13 000 rpm for 20min, 4°C, protein concentration in the supernatant was measured by BC assay (Uptima, Interchim, France). A standard curve using bovine serum albumin was included in each assay.

### Caspase activity

DEVDase activity was measured in 25μg proteins with the fluorometric CaspACE assay system (Promega, WI). To determine the specificity of the assay, measurements were done in presence of caspase specific inhibitor DEVD-CHO [38].

### LDH activity

LDH activity was measured using the Roche diagnostic kit (3004732122) on a Cobas 6000 (Roche Diagnostics GmbH, D-68298, Mannheim). This assay is based on the measure of NADH appearance after transformation of lactate from pyruvate by LDH. Control medium without cells was used as blank.

### Western Blot

Proteins were separated on 12% acrylamide gels and transferred to Immobillon-P transfert membrane (Millipore, MA). After 1h incubation with western blocking reagent (Roche, Germany) the primary antibody was incubated O/N at 4°C. Secondary antibodies were applied for 2h at room temperature. Revelation was performed by chemoluminescence using ECL reagent (Roche, Germany) and the fusion FX7 Imager (Erlangen, Germany).

### RNA expression assay

Cells were grown in 6-wells plate. After treatment, adherent cells were collected and RNA and protein extracted using Nucleospin RNA/Protein kit (Macherey Nagel, Düren, Germany) according to the recommendations. DNAse treatment was included in the protocol.

The quantity and quality of RNA were respectively evaluated using the NanoDrop® ND-1000 spectrophotometer (Nanodrop Technologies, Wilmington, DE) and the Agilent 2100 Bioanalyser (Agilent, Santa Clara, CA). The RNAs extracted were of good quality and the RNA integrity number (RIN) was >9 in all cases. RNA expression was quantified as previously described [40] using the MX3005P, and the mix was the Brilliant II Sybr Green Master Mix (Agilent, CA). Primers sequences are given in Table S1. The housekeeping genes were RPLPO (Ribosomal Protein, Large, PO), and GAPDH.

To insure specificity of the RT-qPCR, an agarose gel electrophoresis was initially performed to check whether a single PCR product was generated and then a melting curve was performed at the end of each RT-qPCR. Linearity and efficiency of the RT-qPCR were checked for each gene with a standard curve of 4 logs prepared with Universal RNA (Stratagene-Agilent, CA). Efficiency was >90% in all cases.

### Statistical analysis

Statistical analyses were performed using GraphPad Prism 5 using unpaired t-Test for group comparisons. Significant differences (p < 0.05) are indicated (*) on figures.

## SUPPLEMENTARY FIGURES AND TABLE




