# Supplementary material for: Bak and Mcl-1 are essential for Temozolomide induced cell death in human glioma
Source: Oncotarget. 2014 Jan 1;5(9):2428–35. doi: 10.18632/oncotarget.1642 (PMC4058016; doi:10.18632/oncotarget.1642)
Supplement: Supplementary file 1 [file oncotarget-05-2428-s001.pdf]

Bak and Mcl-1 are essential for Temozolomide induced cell death in human glioma – Gratas et al

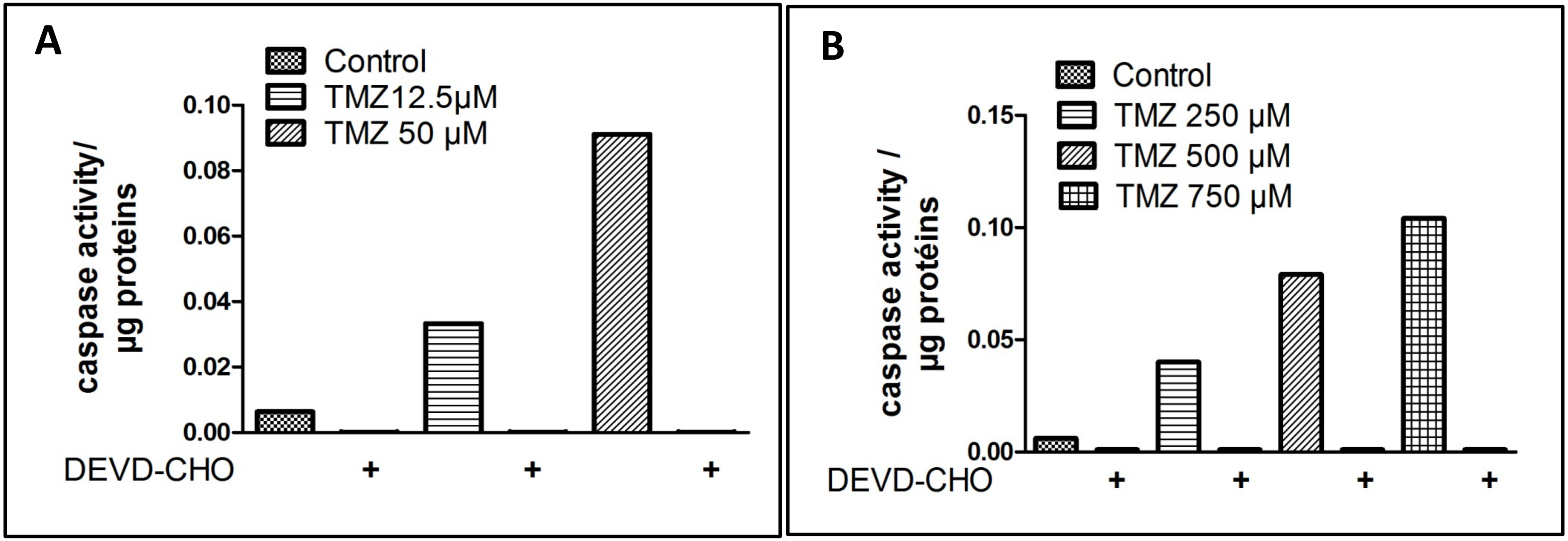

Figure S1: Caspase activity in U251 and LN18 is due to Caspase 3: To check for caspase 3 activity, the inhibitor DEVD-CHO was added in the caspase assay, in a duplicate well. In all cases, the caspase activity was completly abolished. A) U251 cell line B) LN18 cell line.

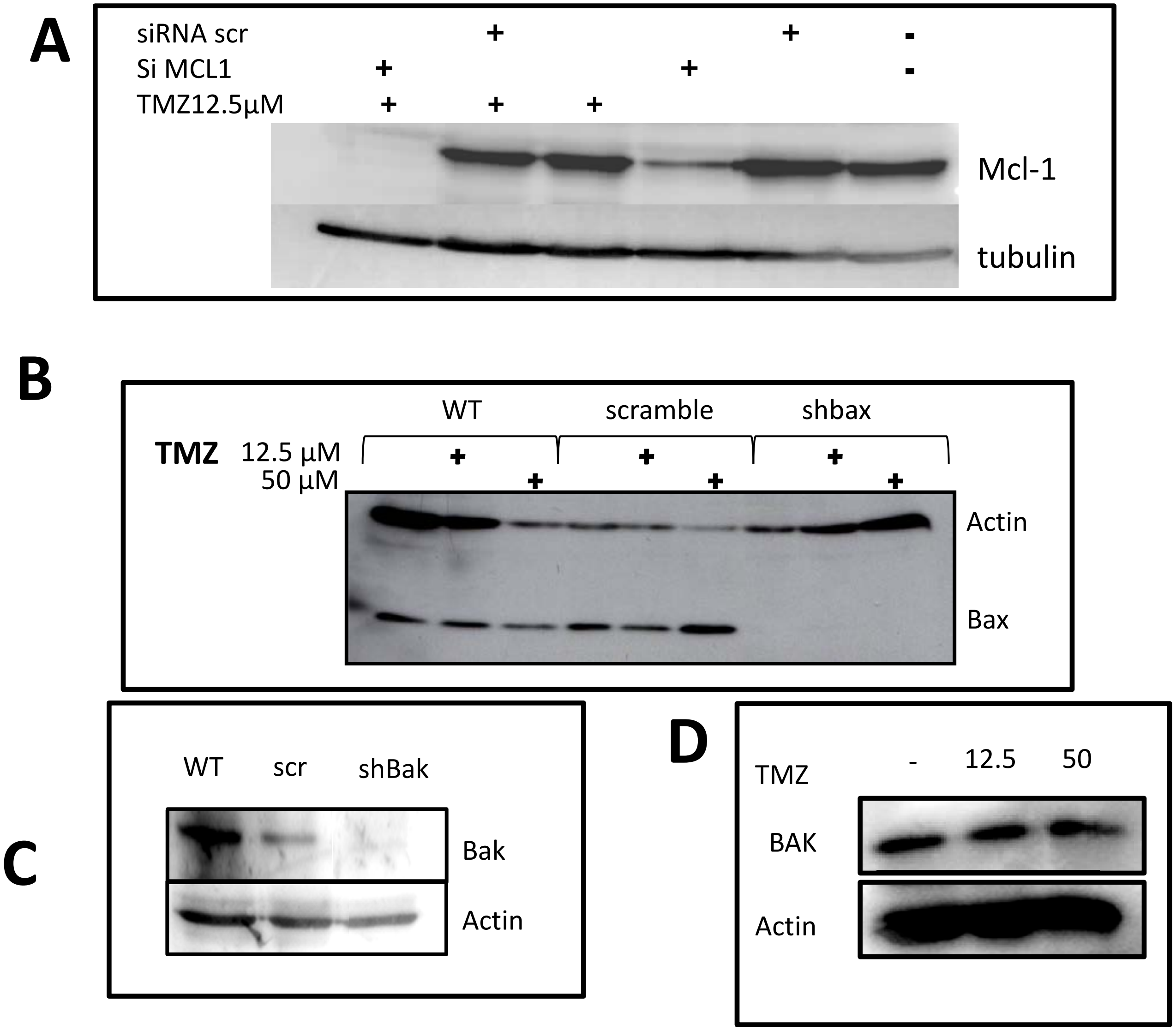

Figure S2: Inhibition of Mcl-1, Bax and Bak expression in U251 cells.
Proteins were extracted from cells and immune-blots were performed, with respective antibodies, on 25µg proteins to check inhibition of protein . (A) Cells transfected with scramble or si-Mcl-1 (B) Cells transduced with scramble or sh-Bax (C) Cells transduced with scramble or sh-Bak. (D) Effect of TMZ on the expression of Bak in U251 cell line

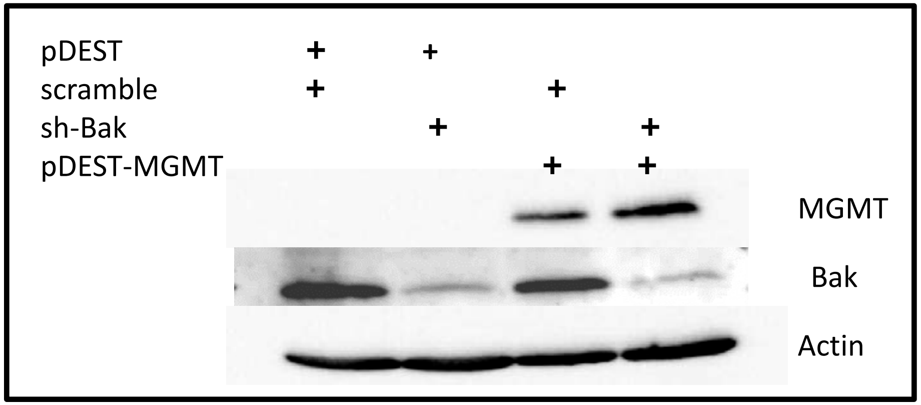

Figure S3: Measure of the expression of MGMT in U251 cell line transfected with pDest alone, pDest-MGMT and treated with sh-scramble, sh-Bax or sh-Bak.

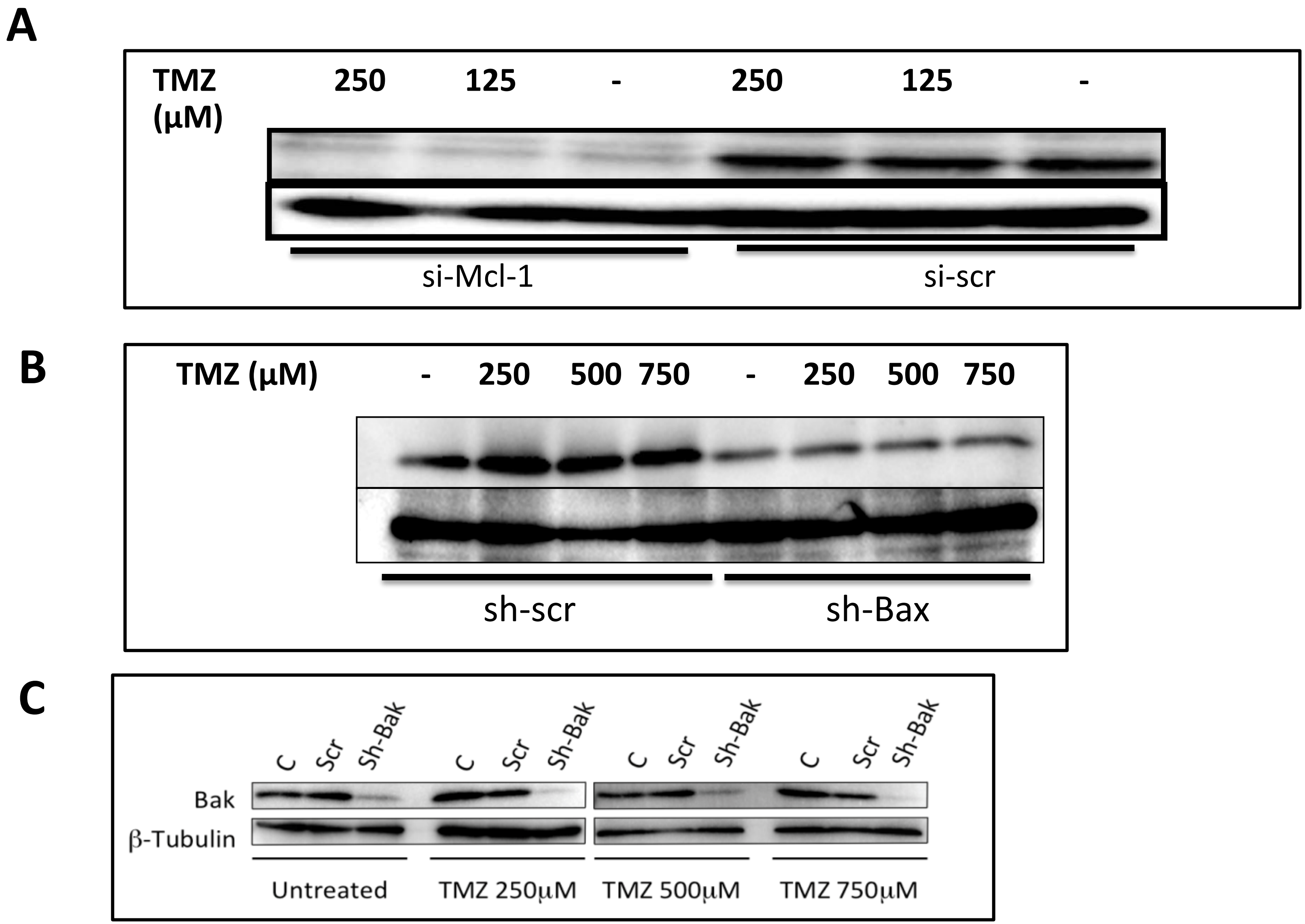

Figure S4: Inhibition of Mcl-1, Bax and Bak expression in LN18 cells.
Proteins were extracted from cells and immune-blots were performed, with respective antibodies, on 25µg proteins to check inhibition of protein . (A) Cells transfected with scramble or si-Mcl-1 (B) Cells transduced with scramble or sh-Bax (C) Cells transduced with scramble or sh-Bak. Please note that the treatment with TMZ did not alter the expression of the proteins.
