# Supplementary material for: Bak and Mcl-1 are essential for Temozolomide induced cell death in human glioma
Source: Oncotarget. 2014 Jan 1;5(9):2428–35. doi: 10.18632/oncotarget.1642 (PMC4058016; doi:10.18632/oncotarget.1642)
Supplement: Supplementary file 2 [file oncotarget-05-2428-s002.pdf]

Bak and Mcl-1 are essential for Temozolomide induced cell death in human glioma – Gratas et al

Table S1: Primers sequences

Gene Name	Sequences
Bak	
	Fow : TATAATACCCTTGTGAGAGCCC<br>Rev : AATCCCTGAGAGTCCAACTG
Bax	Fow : ACCGTGACCATCTTTGTG<br>Rev : AAAACACAGTCCAAGGCA
Bcl-2	Fow : CCTTCTTTGAGTTCGGTGGG<br>Rev : TCTTCAGAGACAGCCAGGAG
Mcl-1	Fow : GCATCGAACCATTAGCAGAA<br>Rev : TGCCACCTTCTAGGTCCTCT
RPLPO	Fow GATTACACCTTCCCACTTGCT<br>Rev : TAGTCAAAGAGACCAAATCCCA
GAPDH	Fow : GAAGATGGTGATGGGATTTC<br>Rev : GAAGGTGAAGGTCGGAGTC
